# Comparative Study on Kernel Quality and Chemical Composition of Ancient and Modern Wheat Species: Einkorn, Emmer, Spelt and Hard Red Spring Wheat

**DOI:** 10.3390/foods10040761

**Published:** 2021-04-02

**Authors:** Jayani Kulathunga, Bradley L. Reuhs, Steve Zwinger, Senay Simsek

**Affiliations:** 1 Cereal Science Graduate Program, Department of Plant Sciences, North Dakota State University, Fargo, ND 58102, USA; jayani.maddakandaged@ndsu.edu; 2 Whistler Center for Carbohydrate Research, Department of Food Science, Purdue University, West Lafayette, IN 47907, USA; breuhs@purdue.edu; 3 Carrington Research Extension Center, North Dakota State University, Carrington, ND 58421, USA; steve.zwinger@ndsu.edu

**Keywords:** ancient wheat, einkorn, emmer, spelt, hulled wheats

## Abstract

Hulled wheat species are often used as whole grains in processing, and have been attracting attention in the last 20 years in the food industry. Whole wheat flour of hulled wheat can be used in the food industry for value addition. This study was conducted to evaluate the kernel quality and chemical composition of the whole grain flour of hulled wheats as a preliminary approach to use these species for value addition. The experimental design was separate, randomized complete block designs for einkorn, emmer, and spelt, with four field replicates. According to the results, significant differences (*p* < 0.05) were observed in kernel quality traits, such as test weight, 1000 kernel weight, and kernel hardness, compared to hard red spring wheat. The results of the chemical composition revealed that hulled wheats were characterized by significantly lower (*p* < 0.05) protein and higher (*p* < 0.05) crude fat contents compared to whole wheat flour of hard red spring wheat. Among hulled wheats, total dietary fiber content was highest in emmer, followed by einkorn and spelt. In conclusion, the whole wheat flour of einkorn, emmer, and spelt used in this study differ from hard red spring wheat in their kernel quality and chemical composition.

## 1. Introduction

Whole grains consist of intact, ground, flaked, or cracked caryopsis, including starchy endosperm, germ, and bran. They play an important role in reducing the risk of cardiovascular diseases and type 2 diabetes, and play a protective role in body weight management, certain types of cancer, and gastrointestinal health [1]. Einkorn, emmer, and spelt are the major types of ancient wheat species that were consumed by people as whole grains for centuries before they were replaced by modern bread wheat. Einkorn (*Triticum monococcum* L. ssp. *monococcum*) is a diploid (2*n* = 2x = 14) hulled wheat carrying the A genome [2]. Cultivated emmer wheat (*Triticum dicoccon* Schrank) is a tetraploid wheat with A and B genomes, and it is a close relative of durum wheat. Moreover, it was one of the basic plants in Neolithic agriculture. Emmer is a minor crop today, cultivated in isolated, marginal areas [3,4]. Spelt is a hexaploid wheat with A, B, and D genomes, and is now cultivated in Europe, Asia, North Africa, and North America [5].

Hulled, or ancient, wheats were the earliest domesticated wheats by mankind, and are the ancestors of modern wheats. Their cultivation drastically decreased during the 1960s due to their low yield and presence of hull, which makes processing difficult. However, the increasing demand for a healthy and equilibrated diet led to the rediscovery of these grains. Therefore, the exploitation of hulled wheat species has become an important factor to further drive consumer trends, as they can satisfy emotionally driven trends created by people [6].

Spelt has occupied a niche market in North America and Europe in the natural, organic, health, and specialty food markets [7]. Moreover, the necessity of food diversification, and plant breeding directed towards improving the nutritional quality of crops, has led to a renewed interest in these species [8]. This increased demand requires information about the agronomic performance and quality traits of einkorn, emmer, and spelt [6]. Definitive comparisons of these species with modern bread wheat are rare due to the difference in farming systems [9]. Einkorn, emmer, and spelt are usually dedicated as primary crops in organic or small-area farms, and are generally tolerant of less advanced cultivation technologies. In contrast, common wheat is the typical industrial raw material, and it is usually cultivated in large production farms. Therefore, results can also be affected by environmental factors [10]. Most of the studies are difficult to compare because the samples were cultivated in different areas and harvest years, were fertilized differently, and the grains were milled to white or wholemeal flours [11]. The samples used in this study were from a field experiment conducted in the same year and location, with identical environmental conditions.

The objective of this study was to evaluate and compare the kernel quality, microstructure of kernels, and nutritional value of hulled wheat with hard red spring wheat as a preliminary approach for value addition. It is important to explore different genotypes of ancient wheat for their nutritional value, to provide updated information on these wheat species.

## 2. Materials and Methods

### 2.1. Materials

Ten genotypes from three different hulled wheat species namely einkorn (TM23, WB Apline, PI538722), emmer (Vernal, Lucille, ND common, Yaroslav), and spelt (CDC Zorba, 94-288, SK3P) were used in this study. Eight genotypes of hard red spring (HRS) wheat (SY Ingmar, Barlow, Elgin-ND, Linkert, Glenn, Rollag, ND Vitpro, Lang-MN) that were cultivated at the same location were used, along with hulled wheat, to compare the results of hulled wheat. All the chemicals and reagents were of analytical grade.

### 2.2. Methods

#### 2.2.1. Sample Preparation

Einkorn, emmer, spelt, and hard red spring wheat samples were cleaned by passing through a dockage tester (XT5 3/03 (Carter Day International, Minneapolis, MN, USA)). Cleaned einkorn, emmer, and spelt samples were dehulled using a laboratory impact dehuller in the pilot plant of North Dakota State University. Next, the samples were milled on a Quadramat Junior laboratory mill (C.W. Brabender Instrument, Inc., South Hackensack, NJ, USA) by removing the sieve to produce whole wheat flour. The conditions in the milling laboratory were maintained as follows: temperature (22 ± 1 °C) and relative humidity (65% ± 3%). All samples were stored in sealed plastic bags and placed in the cooler at 4 °C until analysis.

#### 2.2.2. Kernel Quality Traits

Kernel quality traits such as test weight, 1000 kernel weight (TKW), kernel hardness index, and kernel size distribution were evaluated for all the samples. The test weight of kernels was measured by using the (American Association of Cereal Chemists International) AACCI Method 55-10.01. Thousand kernel weight was determined by removing all dockage, shrunken and broken kernels, and other foreign material from wheat samples. A mechanical seed counter (Seedburo Equipment Co., Chicago, IL, USA) was used to count ten grams of wheat, and the number of kernels in ten grams was converted to 1000 kernel weight. The kernel hardness index was determined as described by using a single kernel characterization system [12]. Kernel size distribution was determined by sieving the wheat on Tyler No. 7 (2.92 mm openings) and Tyler No.9 (2.24 mm openings) sieves. Large, medium, and small kernels, which were the amounts of overs from the Tyler No. 7 and Tyler No. 9 sieves, and remainders, respectively, were designated as percentages of the total amount of wheat.

#### 2.2.3. Scanning Electron Microscopy (SEM) Images of the Transverse Section of the Wheat Kernel, Endosperm, and Bran Layers

Kernels were cut vertically using a razor blade, and transverse sections were attached to cylindrical aluminum mounts using colloidal silver paste (SPI Supplies, West Chester, Pennsylvania) and sputter-coated (Model SCD 030, Balzers, Liechtenstein) with gold palladium. Images of the transverse section of the kernel, endosperm, and outer layers were obtained with a JEOL JSM-6490LV scanning electron microscope (JEOL USA, Peabody, MA, USA), operating at an accelerating voltage of 15 kV.

#### 2.2.4. Proximate Composition of Whole Wheat Flour

The total starch, protein, crude fat, and ash content of whole wheat flour was analyzed by AACCI methods of 76-13.01, 46-19.01, 30-25.01, and 08-01.01, respectively.

#### 2.2.5. Fatty Acid Composition of Whole Wheat Flour

The fatty acid composition was analyzed by gas chromatography (AACCI method 58-18.01).

#### 2.2.6. Dietary Fiber Content of Whole Wheat Flour

Dietary fiber analysis was performed according to the Association of Official Agricultural Chemists (AOAC) 2011.25/32-50.01 method, using an ANKOM^TDF^ automated dietary fiber analyzer. The total dietary fiber content was calculated as a sum of insoluble and soluble dietary fiber fractions.

### 2.3. Statistical Analysis

The field design for the hulled wheat was a separate randomized complete block design (RCBD) with four field replicates for each genotype. Data were subjected to analysis of variance using the ‘’General Linear Model’’ (GLM) procedure in Statistical Analysis System 9.4 (SAS Institute, Cary, NC, USA). Differences were considered significant when the probability value *p* was lower than 0.05. A least significant difference (LSD) with a 5% significance level was used to declare differences. Data were presented as the means, with standard deviation averaged across four field replicates.

## 3. Results and Discussion

### 3.1. Kernel Quality of Hulled Wheat

Mean values for the kernel quality traits of hulled wheat species and HRS are shown in Table 1. Significant differences (*p* < 0.05) were observed in test weight, 1000 kernel weight, and the hardness index of hulled wheats compared to HRS. Test weight is used as an indicator of grain quality, and as a measure of grain bulk density. It is used to determine the price of wheat during trading [13]. Among hulled wheats, the highest test weight was for spelt (94-288 genotype), while the lowest was for emmer (Yaroslav). This implies a good flour extraction rate in spelt among the hulled wheats.

Thousand kernel weight (TKW) is an indicator of wheat milling value [14]. The highest 1000 kernel weight value was reported for spelt (44 g), while the lowest was for einkorn (28 g) when compared to HRS (Table 1). Lower TKW values affect the acceptance of einkorn by millers. Similar values were reported for einkorn in several studies [15,16,17]. The TKW of emmer ranged from 32.9 to 34.0 g, and these values seemed to be low compared to the values reported in other studies [18].

Similarly, the TKW values of these spelt samples were low when compared with a previous study done by Oberkulmer Rotkorn and Rouquin using samples grown in Europe [19]. This could be due to differences in climate, agronomic practices, genotypes, and growth location.

The hardness index is a major factor that can influence kernel milling properties [20]. Friabilin protein content is one of the most important factors, which is used to classify hardness into two classes: high and low [21]. The kernel hardness index values of einkorn and spelt had significant differences (*p* < 0.05) when compared to HRS (Table 2). No significant differences were observed between emmer and HRS. Einkorn can be categorized as extra soft wheat since the hardness index is below 50, while emmer can be categorized as hard wheat similar to common bread wheat. Both soft (CDC Zorba and SK3P) and hard (94-288) genotypes were found in spelt; however, higher hardness values have been reported in one study for einkorn, while similar hardness values have been reported for some genotypes of spelt [16]. Harder wheat needs to be tempered and conditioned for a longer period of time compared to softer wheat, to obtain refined flour. Therefore, lower hardness values imply that einkorn and spelt (except 94-288) do not require tempering before milling.

According to the results of kernel sizing (Table 1), significant differences (*p* < 0.05) were observed in hulled wheat compared to HRS. The majority of kernels in einkorn and emmer were medium-sized (90%), while in HRS, only about 60% of kernels were medium-sized. Interestingly, 80% of kernels were large in one genotype of spelt (SK3P), while the other two genotypes of spelt had larger kernels ranging between 35 and 44%. These results revealed that there could be differences in kernel size among genotypes of spelt grown under the same environmental conditions. The results of kernel quality traits can be used to design and manage breeding strategies for grain quality improvement. Moreover, they are useful for the milling industry.

### 3.2. Microstructure of Hulled Wheat Kernels by Scanning Electron Microscopy (SEM)

**Figure 1:**
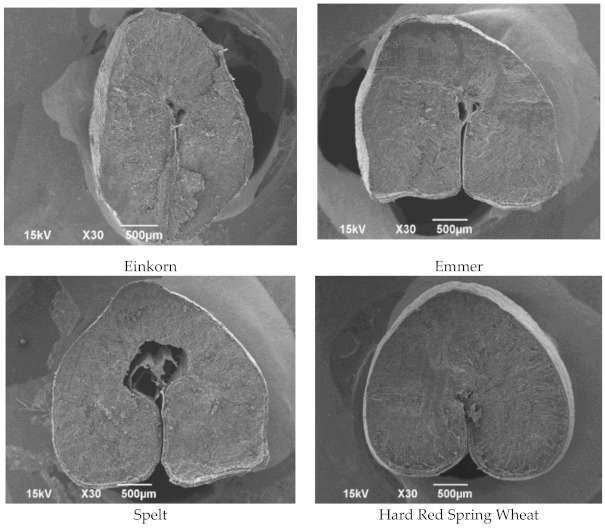
The microstructure of the transverse section of wheat kernels. (This material is based upon work supported by the National Science Foundation under Grant Nos. 0619098, 0821655, 0923354, and 1229417. Any opinions, findings, conclusions, or recommendations expressed in this material are those of the author(s), and do not necessarily reflect the views of the National Science Foundation.)

**Figure 2:**
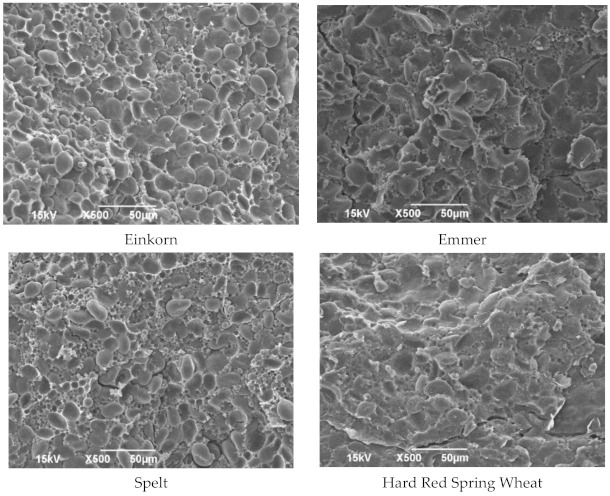
The microstructure of the endosperm of wheat kernels. (This material is based upon work supported by the National Science Foundation under Grant Nos. 0619098. 0821655, 0923354, and 1229417. Any opinions, findings, conclusions, or recommendations expressed in this material are those of the author(s), and do not necessarily reflect the views of the National Science Foundation.)

**Figure 3:**
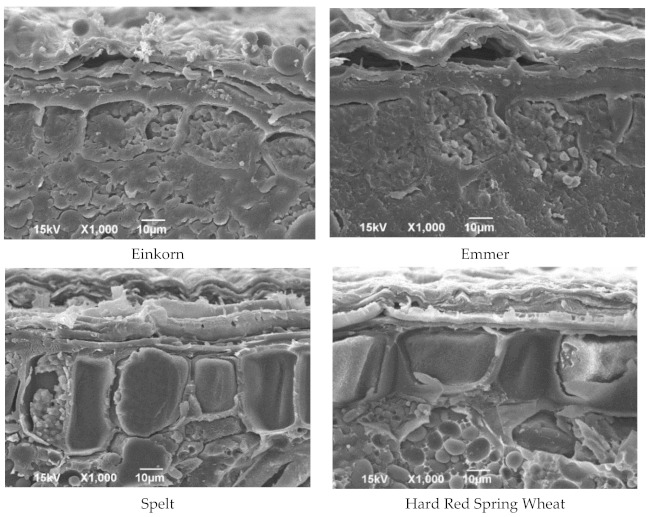
The microstructure of bran layers. (This material is based upon work supported by the National Science Foundation under Grant Nos. 0619098, 0821655, 0923354, and 1229417. Any opinions, findings, conclusions, or recommendations expressed in this material are those of the author(s), and do not necessarily reflect the views of the National Science Foundation.)

The microstructure of hulled wheat kernels is important, as there were pronounced differences in kernel hardness that were discussed above under quality traits (Section 3.1). Therefore, SEM images of the transverse section of kernels, endosperm structure, and bran layers were obtained, and are shown in Figure 1, Figure 2 and Figure 3, respectively. The transverse section of the einkorn kernel is unique and elongated,different in shape compared to emmer, spelt, and HRS (Figure 1). The shape could be another factor that can affect the packing efficiency of wheat, thus influencing test weight [22]. This could be a possible reason for the differences in test weights among hulled wheat species.

Mature wheat (*Triticum aestivum* L.) endosperm contains two types of starch granules: large (10–35 µm) A-type and small (1–10 µm) B-type [23]. Similarly, discrete and two separate types of starch granules were observed in einkorn (Figure 2). Furthermore, large granules are lenticular in shape, while small granules are spherical in shape. Moreover, starch granules are loosely packed, and it was observed that they were less embedded in a partial presence of the protein matrix. This was opposed to the features observed in emmer and HRS (Figure 2). The endosperm of emmer (Figure 2) showed densely packed compact starch granules that were firmly cemented by a protein matrix. Unlike einkorn, large and small starch granules are completely embedded in the protein matrix. Moreover, a much more complete protein matrix was observed. Emmer is similar to HRS wheat, as it showed direct physical attachment connection between starch and protein. Starch granules of spelt wheat were observed to be noticeably less firmly embedded in the internal matrix protein structure than the starches of the harder emmer and HRS wheat varieties, but more firmly embedded than einkorn (Figure 2). Similar results were observed in another study related to the endosperm of einkorn, emmer, and spelt [24].

The aleurone and outer layers of einkorn, emmer, spelt, and HRS are shown in Figure 3. Einkorn is characterized by a thinner pericarp and cube-shaped aleurone cells. The presence of a thinner pericarp, incomplete protein matrix, and air pockets would be possible reasons for the softness of einkorn kernels, as noticed in this study. Cereals with small-sized starch granules and thinner cell walls are more easily digested and absorbed compared to cereals with large starch granules and thick cell walls, due to their better bio-accessibility to amylase [25]. In emmer, the aleurone layer is characterized by thick cell walls and cube-shaped cells (Figure 3). Spherical protein bodies are visualized inside aleurone cells. More elongated aleurone cells were observed in spelt compared to other species (Figure 3). However, a clear sub-aleurone layer was not observed as in HRS. These results suggested that differences in kernel hardness that exist among einkorn, emmer, spelt and HRS are related to the morphological differences in the kernel structure.

### 3.3. Chemical Composition of Whole Wheat Flour of Hulled Wheats

The proximate compositions of the einkorn, emmer, and spelt whole wheat flour were analyzed and compared to eight genotypes of HRS whole wheat flour samples (Table 2). The moisture content values in the whole wheat flour of hulled wheats varied between 8.8 and 9.5% (Table 2), while the whole wheat flour of the HRS genotypes had values around 10.0–10.5%. Moisture content is not a wheat-grade determinant, but is important for providing information used for pricing the commodity, and is essential information when storing or processing the wheat. Significant differences (*p* < 0.05) were reported in the moisture content of hulled wheat flour compared to HRS whole wheat flour. This could be due to the differences in harvest dates of different wheat species.

The ash content ranged from 2.0 to 2.3% and 2.0 to 2.2% for hulled wheats and HRS, respectively. The ash content values in the hulled wheat flour were similar to those previously reported in different studies [15,17].

The whole wheat flour of hulled wheats showed protein contents between 13.6 and 15.8% (Table 2), while for HRS, the protein content was between 15.9 and 18.4% (Table 2). As it was observed, significantly lower (*p* < 0.05) protein contents were found in hulled wheats compared to HRS. Lower protein contents (11.1–11.6%) were reported for the whole wheat flour of einkorn, emmer, and spelt in a study conducted using samples grown in Germany [26]. However, several studies revealed that the protein content in the whole wheat flour of ancient wheat is commonly superior to that of its modern counterpart [3,17]. The difference in protein contents can be due to differences in fertilization levels [27]. These differences could also be due to agronomic practices, soil fertility, disease control, and weed control. Thus, the evaluation of different farming practices, such as the use of alternative growth regulators and different fertilization levels, are of high interest and require further research.

The starch content of wheat is related to the yield, but also to the quality properties of different wheat-based foods [28]. A significantly higher (*p* < 0.05) starch content was observed for emmer, but the difference was not significant for einkorn or spelt compared to HRS. The starch content in both hulled wheats and HRS varied from 61.2 to 66.9% and 60.5 to 65.1%, respectively. The values for einkorn and emmer were similar to the values reported in other research studies. Total starch contents for einkorn and emmer were reported as 62.3–65.0% [16]. Higher values were observed for emmer (66%) when compared to bread wheat, while lower values were observed for einkorn and spelt. However, several studies reported either a comparable or even lower starch content for hulled wheats compared with that of bread wheat [15,29].

Crude fat content is a minor component of wheat grain compared to starch and protein, ranging from 0.57 to 1.44% and 1.25 to 2.53% for hard red spring wheat and hulled wheats, respectively (Table 2). Hulled wheat had a higher crude fat content than common bread wheat, and these findings are consistent with the findings of another study. [30]. Significantly higher (*p* < 0.05) crude fat contents were reported for hulled wheat flour when compared to HRS whole wheat flour. Moreover, significant differences (*p* < 0.05) in the fat content were also observed between genotypes for spelt. It is believed that genetic predisposition has the biggest influence on lipid content in wheat. Furthermore, the lipid content in different genotypes of wheat grown in the same environment varied more than when the same wheat genotype was grown in different environmental conditions [31].

The AOAC 2011. 25/32-50.01 method was used to quantify each of the components of dietary fiber, including fructo-oligosaccharides. Total dietary fiber content has been underestimated by most methods used, as these methods do not quantify fructo-oligosaccharides [32]. To the best of our knowledge, this study is the first publication on the use of the newly developed integrated total dietary fiber assay AOAC 2011.25/32-50.01 that evaluated the dietary fiber variation in ancient wheat species. In this study, significantly lower dietary fiber values were reported in einkorn compared to HRS, while comparable values were reported in emmer. However, lower values were reported for hulled wheat species in other studies [1]. This could be due to differences in the method of quantification.

### 3.4. Fatty Acid Composition of Whole Wheat Flour of Hulled Wheats

The fatty acid composition of einkorn, emmer, spelt and HRS is shown in Table 3. Significant differences (*p* < 0.05) were observed for einkorn, emmer and spelt in the content of palmitic acid, linoleic, linolenic, and oleic acid when compared to HRS.

One genotype of spelt was characterized by the highest concentration of linoleic acid (62.5%) among hulled wheats, while einkorn had the highest contents of palmitoleic acid (0.23%) and oleic acid (27%) among hulled wheats. Interestingly, hulled wheats were low in saturated fatty acids, such as palmitic acid, and they were higher in monounsaturated fatty acids, such as oleic acid, compared to HRS. However, polyunsaturated fatty acids, such as linoleic and linolenic acids, were reported to be low in einkorn, emmer, and spelt, compared to HRS. Similar results were observed for einkorn in other studies [33]. The results of fat content and fatty acid composition are important for determining nutritional value and the long-term storage ability of cereal-based foods, although values are low.

## 4. Conclusions

Significant differences (*p* < 0.05) were observed in the test weight and grain hardness of hulled wheat, which could be explained by the shape of the kernel and the microstructure of endosperm. Einkorn was identified as extra soft-textured kernels; in contrast, emmer was a hard-type wheat, similar to hard red spring wheat. Both medium-soft and hard genotypes were observed in spelt. The chemical composition of hulled wheats revealed they have significantly higher (*p* < 0.05) crude fat contents. Therefore, further studies should investigate the stability of lipid components during storage and while in food products. In addition, aspects that need to be addressed in any such comparative study should include genotype and location interactions, and other required phenotypic assessments, through multiple-year and multiple-location trials.

## Figures and Tables

**Table 1:** Kernel quality traits of einkorn, emmer, spelt and hard red spring wheat.

Wheat Species	Genotype	Test Weight (kg/hL)	Average	1000 Kernel Weight (g)	Average	Large Kernel Content (%)	Average	Medium Kernel Content (%)	Average	Small Kernel Content (%)	Average	Hardness Index	Average
Einkorn	TM 23	72.4	73.2 ± 1.1 b	30.9	29.2 ± 1.5 c	2.8	3.8 ± 2.8 c	92.4	92.9 ± 3.9 a	4.0	3.0 ± 1.1 b	1.8	2.2 ± 0.4 c
	WB Apline	74.5		28.8		1.7		97.0		1.8		2.4	
	PI 538722	72.7		28.0		7.0		89.3		3.1		2.5	
Emmer	Vernal	71.3	70.4 ± 1.3 c	33.8	33.6 ± 0.5 b	3.4	2.7 ± 0.8 c	91.4	91.9 ± 1.2 a	4.9	5.2 ± 2.4 a	73.8	74.4 ± 0.8 a
	Lucille	71.4		34.0		3.1		93.4		3.4		75.6	
	ND common	70.2		32.9		2.8		90.7		6.0		74.4	
	Yaroslav	68.7		33.6		1.6		92.1		6.3		73.8	
Spelt	CDC Zorba	70.5	72.9 ± 2.3 b	35.1	38.3 ± 4.9 a	44.0	52.9 ± 23.3 a	54.2	34.85 ± 22.6 c	1.7	0.9 ± 0.8 c	24.4	32.7 ± 20.4 b
	94-288	75.0		35.8		35.4		64.2		0.8		56.0	
	SK3P	73.2		44.0		79.3		21.0		0.1		17.8	
HRS	Sy Ingmar	79.8	81.0 ± 1.0 a	33.9	33.9 ± 1.5 b	31.9	33.7 ± 2.6 b	67.0	65.2 ± 2.4 b	1.2	1.1 ± 0.4 c	68.3	74.9 ± 5.3 a
	Barlow	81.3		32.8		31.4		67.4		0.9		79.8	
	Elgin-ND	79.4		33.7		33.9		64.4		1.7		75.3	
	Linkert	80.6		36.6		38.4		61.0		0.6		66.1	
	Glenn	82.1		34.3		32.7		66.0		1.4		77.4	
	Rollag	81.2		34.3		36.6		62.2		1.2		78.0	
	ND Vitpro	81.9		34.4		33.3		66.2		0.4		73.1	
	Lang-MN	81.8		31.5		31.4		67.1		1.5		80.8	

HRS–Hard Red Spring. Mean values across field replicates are presented in Table 1 (*n* = 4). Means with the same letter in the same column are not significantly different (*p* < 0.05).

**Table 2:** Chemical composition of whole wheat flour of einkorn, emmer, spelt and hard red spring wheat.

Species	Genotype	Moisture (%)	Average	Ash (%)	Average	Protein (%)	Average	Total Starch (%)	Average	Crude Fat (%)	Average	Total Dietary Fiber Content (%)	Average
Einkorn	TM 23	9.0	9.0 ± 0.1 c	2.2	2.2 ± 0.1 a	15.4	14.6 ± 0.8 c	62.1	62.2 ± 0.6 b	2.3	2.3 ± 0.2 a	13.0	15.1 ± 2.3 c
	WB Apline	9.0		2.2		13.9		62.8		2.5		14.8	
	PI 538722	9.1		2.1		14.5		61.7		2.1		17.5	
Emmer	Vernal	9.4	9.4 ± 0.1 b	2.3	2.2 ± 0.1 a	15.2	14.5 ± 0.7 c	64.3	65.9 ± 1.2 a	2.0	2.1 ± 0.2 a	15.0	19.1 ± 3.1 a
	Lucille	9.5		2.3		15.0		66.9		2.0		20.7	
	ND common	9.5		2.1		14.2		66.6		2.0		18.4	
	Yaroslav	9.3		2.1		13.6		65.8		2.3		22.2	
Spelt	CDC Zorba	8.8	8.9 ± 0.2 c	2.2	2.1 ± 0.1 a	14.6	15.2 ± 0.6 b	61.2	61.6 ± 0.4 b	1.7	1.6 ± 0.3 b	15.6	17.3 ± 1.5 ac
	94-288	9.1		2.0		15.1		61.8		1.3		17.8	
	SK3P	8.9		2.1		15.8		61.9		1.9		18.5	
HRS	Sy Ingmar	10.5	10.4 ± 0.2 a	2.1	2.2 ± 0.1 a	18.4	17.3 ± 0.8 a	61.4	62.3 ± 1.7 b	1.0	1.1 ± 0.3 c	18.8	19.2 ± 1.8 a
	Barlow	10.4		2.1		16.6		65.1		0.7		17.6	
	Elgin-ND	10.3		2.2		16.9		60.5		1.2		20.7	
	Linkert	10.0		2.2		17.5		62.9		1.4		21.3	
	Glenn	10.4		2.2		17.3		64.3		1.3		19.1	
	Rollag	10.3		2.1		17.6		61.5		1.4		21.6	
	ND Vitpro	10.4		2.3		17.9		62.0		0.9		17.4	
	Lang-MN	10.8		2.0		15.9		60.5		0.6		17.4	

HRS—Hard Red Spring. Mean values across field replicates are presented in Table 2 (*n* = 4). The ash content, protein content, total starch content, crude fat content, and total dietary fiber content values are expressed on dry weight basis. Mean values with the same letter in the same column are not significantly different (*p* < 0.05).

**Table 3:** Fatty acid composition of whole wheat flour of einkorn, emmer, spelt and hard red spring wheat.

**Wheat Species**	**Genotype**	**PAL**	**AVG**	**PAO**	**AVG**	**MAR**	**AVG**	**STE**	**AVG**	**OLE**	**AVG**	**VAC**	**AVG**
Einkorn	TM 23	15.0	15.4 ± 0.4 c	0.2	0.2 ± 0.0 a	0.3	0.2 ± 0.1 a	1.2	1.1 ± 0.1 a	28.0	27.8 ± 0.2 a	1.1	1.1 ± 0.1 a
	WB Apline	15.4		0.2		0.2		1.0		27.8		1.2	
	PI 538722	15.7		0.2		0.2		1.2		27.6		1.1	
Emmer	Vernal	17.6	17.6 ± 0.1 b	0.2	0.2 ± 0.1 a	0.2	0.2 ± 0.0 a	1.6	1.5 ± 0.1 a	27.1	25.8 ± 1.2 b	0.9	1.0 ± 0.1 a
	Lucille	17.7		0.2		0.2		1.6		26.4		0.9	
	ND common	17.6		0.3		0.2		1.5		24.7		1.0	
	Yaroslav	17.6		0.2		0.2		1.3		24.9		1.1	
Spelt	CDC Zorba	16.9	17.5 ± 1.0 b	0.2	0.3 ± 0.1 a	0.2	0.2 ± 0.1 a	0.6	1.0 ± 0.4 a	25.1	21.4 ± 4.3 c	1.1	1.1 ± 0.0 a
	94-288	18.7		0.3		0.3		1.2		16.7		1.1	
	SK3P	16.9		0.3		0.2		1.3		22.3		1.1	
HRS	Sy Ingmar	19.9	19.4 ± 0.5 a	0.2	0.2 ± 0.0 a	0.2	0.2 ± 0.0 a	1.2	1.2 ± 0.0 a	16.2	15.0 ± 1.0 d	1.0	1.0 ± 0.0 a
	Barlow	20.5		0.2		0.2		1.1		12.7		1.0	
	Elgin-ND	18.9		0.2		0.2		1.2		15.8		1.0	
	Linkert	19.2		0.2		0.2		1.2		14.8		1.0	
	Glenn	19.1		0.2		0.2		1.2		15.3		1.0	
	Rollag	19.1		0.2		0.2		1.2		15.1		1.0	
	ND Vitpro	19.1		0.2		0.2		1.2		15.2		1.0	
	Lang-MN	19.1		0.2		0.2		1.2		15.1		1.0	
**Wheat Species**	**Genotype**	**LIO**	**AVG**	**LIN**	**AVG**	**GON**	**AVG**	**EDA**	**AVG**	**HAL**	**AVG**	**NER**	**AVG**
Einkorn	TM 23	54.0	54.2 ± 0.4 c	3.6	3.3 ± 0.3 b	1.5	1.5 ± 0.1 a	0.1	0.1 ± 0.0 b	0.1	0.0 ± 0.1 a	0.2	0.2 ± 0.0 a
	WB Apline	54.7		3.2		1.6		0.1		0.0		0.2	
	PI 538722	54.0		3.1		1.4		0.1		0.0		0.2	
Emmer	Vernal	53.0	54.4 ± 1.4 c	3.4	3.3 ± 0.1 b	1.4	1.3 ± 0.1 b	0.1	0.1 ± 0.0 b	0.1	0.1 ± 0.1 a	0.2	0.2 ± 0.1 a
	Lucille	53.3		3.4		1.3		0.1		0.1		0.1	
	ND common	55.2		3.2		1.3		0.1		0.1		0.1	
	Yaroslav	55.9		3.3		1.3		0.1		0.0		0.2	
Spelt	CDC Zorba	56.6	59.5 ± 3.0 b	2.5	2.9 ± 0.4 c	1.2	0.9 ± 0.3 c	0.1	0.1 ± 0.0 b	0.0	0.1 ± 0.1 a	0.1	0.1 ± 0.0 b
	94-288	62.5		3.2		0.7		0.1		0.1		0.1	
	SK3P	59.4		3.0		0.7		0.1		0.1		0.1	
HRS	Sy Ingmar	61.8	62.4 ± 0.6 a	3.9	3.8 ± 0.1 a	0.7	0.7 ± 0.1 d	0.2	0.2 ± 0.0 a	0.1	0.1 ± 0.0 a	0.1	0.2 ± 0.2 a
	Barlow	63.1		4.0		0.7		0.2		0.1		0.2	
	Elgin-ND	61.4		3.8		0.7		0.2		0.1		0.1	
	Linkert	63.1		3.7		0.6		0.2		0.1		0.2	
	Glenn	62.3		3.8		0.7		0.2		0.1		0.2	
	Rollag	62.7		3.7		0.6		0.2		0.1		0.2	
	ND Vitpro	62.5		3.7		0.7		0.2		0.1		0.2	
	Lang-MN	62.6		3.7		0.6		0.2		0.1		0.2	

AVG: average across species; PAL: palmitic (16:0); PAO: palmitoleic (16:1); MAR: margaric (17:0); STE: stearic (18:0); OLE: oleic (18:1); VAC: vaccenic (18:1); LIO: linoleic (18:2*n*6); LIN: linolenic (18:3*n*3); GON: gonodic (20:1*n*9); EDA: eicosadienoic acid (20:2); HAL: homo-α-linolenic (20:3*n*3); NER: nervonic (24:1*n*9). Values are expressed as the percent of total fat. Mean values with the same letter in the same column are not significantly different (*p* < 0.05).

## Data Availability

Data available on request.
